# On the Wasting Diseases of Infants and Children

**Published:** 1871-05

**Authors:** 


On the Wasting Diseases of Infants and Children. By Eustace Smith, M.D., London. Second American, from the second revised and enlarged English edition. Philadelphia: Henry C. Lea, publisher.

This is a small volume of 253 pages, devoted to the consideration of a very serious and important group of diseases, thus: "Atrophy from Insufficient Nourishment," "Chronic Diarrhœa," "Chronic Vomiting," "Rickets," "Inherited Syphilis," "Mucous Disease," "Worms," "Chronic Tuberculosis," "Chronic Pulmonary Phthisis," "Tuberculization of Glands," "Bronchial Phthisis," and a concluding chapter on the "Diet of Children."

